# Trypsin-independent porcine epidemic diarrhea virus US strain with altered virus entry mechanism

**DOI:** 10.1186/s12917-017-1283-1

**Published:** 2017-11-25

**Authors:** Yunjeong Kim, Changin Oh, Vinay Shivanna, Richard A. Hesse, Kyeong-Ok Chang

**Affiliations:** Diagnostic Medicine and pathobiology, College of Veterinary Medicine, Kansas State University, 1800 Denison Avenue, Manhattan, KS 66506 USA

**Keywords:** Porcine epidemic diarrhea virus, Coronavirus, Virus entry, Attenuation, Live attenuated vaccine, trypsin independent, entry

## Abstract

**Background:**

Porcine Epidemic Diarrhea Virus (PEDV) is a coronavirus that infects the intestinal tract and causes diarrhea and vomiting in older pigs or extreme dehydration and death that could reach 100% mortality in neonatal piglets. In the US, the first PEDV outbreaks occurred in 2013 and since then US PEDV strains have quickly spread throughout the US and worldwide, causing significant economic and public health concerns. Currently two conditionally approved vaccines exist in the US, but there is no live attenuated vaccine, which is considered the best option in controlling PEDV by inducing transferrable mucosal immunity to susceptible neonatal piglets. In this study, we passaged an US PEDV isolate under various conditions to generate three strains and characterized their growth and antigenicity in cell culture using various assays including Western blot analysis, serum neutralization assay, sequencing analysis and confocal microscopy. Finally, these strains were evaluated for pathogenicity in nursing piglets (1–4 days old).

**Results:**

One of the PEDV strains generated in this study (designated as PEDV 8aa) is able to replicate in cells without any protease and grows to a high titer of >8 log_10_ TCID_50_/ml in cell culture. Interestingly, replication of PEDV 8aa was severely reduced by trypsin and this correlated with the inhibition of virus attachment and entry into the cells. In neonatal nursing piglets, PEDV 8aa (passage number 70 or 105) was found to be fully attenuated with limited virus shedding.

**Conclusions:**

These results suggest that applying selective pressure during viral passages can facilitate attainment of viral attenuation and that PEDV 8aa warrants further investigation as an attenuated vaccine.

## Background

Porcine epidemic diarrhea virus (PEDV) is a coronavirus that can cause diarrhea and vomiting in the affected pigs with high mortality of up to 100% in neonatal piglets. Since the first report of PEDV case in the UK in 1971 [1], PEDV has spread throughout the EU during the 1970s and 1980s [2, 3]. While PEDV strains are classified into two distinct genogroups (1 and 2) and subgroups within the genogroups (a and b) [4, 5], recent reports suggest more than 2 genogroups may exist in the field [6]. In the last 30 years or so, PEDV genogroup 1 (and more recently genogroup 2) caused outbreaks with extensive economic losses in some Asian countries with up to 80% to 100% morbidity and 50% to 90% fatality in suckling piglets [7–9]. In the US, the first PEDV outbreaks occurred in 2013 [10]. Since then the US PEDV strains that belong to subgroup 2a have quickly spread to the most states as well as Canada and Mexico [4, 11–13]. The US PEDV strains were also reported to have caused outbreaks in Asian and European countries [14–23], raising significant economic and public health concerns worldwide [24, 25]. Modified live attenuated vaccines (MLVs) for PEDV genogroup 1 are available in Asian countries, and they have been the major means to control PEDV [26–29]. However, the genogroup 1 MLVs may not provide effective protective immunity to the circulating subgroup 2a PEDV strains due to the genetic diversity of about 10% in the S1 gene between the genogroups [4, 11, 30]. Currently two conditionally approved vaccines exist in the US: alphavirus-based vaccine (Harrisvacccines) and an inactivated vaccine (Zoetis) [31]. However, MLVs are not yet available for US PEDV strains.

Administration of an MLV, followed by a booster dose of an inactivated vaccine or an MLV in pregnant sows is generally considered an effective measure for controlling PEDV; MLV would effectively prime the immune system of the pregnant sows, especially PEDV naïve sows, for the production of antibodies, which are transferred to neonatal piglets and protect them from viral infections during the most susceptible period (< 2 weeks of age) [26, 32]. In this study, to develop an MLV for US PEDV strains, we isolated an US PEDVstrain and serially passaged the virus under various culture conditions, using trypsin, elastase and glychenodeoxycholic acid (GCDCA), for up to 120 passages in Vero cells. The resulting virus strains, designated as PEDV KD (grown in trypsin), PEDV AA (in elastase) and PEDV 8aa (in GCDCA), were characterized for their growth in cell culture and/or evaluated for attenuation in piglets. After serial passages, PEDV 8aa gained the ability to replicate in cells without any protease, and grew to a high titer of >8 log_10_ tissue culture infectious dose 50 (TCID_50_)/ml in cell culture. Interestingly, the replication of PEDV 8aa was severely reduced by trypsin and this correlated with the inhibition of virus attachment and entry into the cells. While PEDV KD or PEDV AA retained virulence even after passage number 100, PEDV 8aa showed complete attenuation in neonatal piglets after passage number 70. These results warrant further investigation of PEDV 8aa as an MLV candidiate.

## Methods

### Ethics statement

All animal studies were conducted at Life Science Annex, University of Nebraska, Lincoln, in strict compliance with the Animal Welfare Act, PHS Policy and other federal statutes and regulations relating to animals and approved by the Institutional Animal Care and Use Committee at University of Nebraska at Lincoln (Protocol Number:1006). Animals were monitored twice daily for illness such as weight loss, dehydration or loss of mobility and all efforts were made to minize suffering. Animals that show clincal signs were assessed by an attending veterinarian and euthanized using euthanasia solution after being anesthetized in accordance with the regulations.

### Cells, viruses, and reagents

Vero (ATCC®-CCL-81™) cells were obtained from ATCC (Manassas, VA). Cells were maintained in Dulbecco’s minimal essential medium (DMEM) containing 5% fetal bovine serum (FBS) and antibiotics (chlortetracycline [25 μg/ml], penicillin [250 U/ml], and streptomycin [250 μg/ml]). L-1-tosylamide-2-phenylethyl chloromethyl ketone (TPCK)-treated trypsin and GCDCA were purchased from Sigma-Aldrich (St Louis, MO) and elastase from porcine pancreas was obtained from Promega (Madison, WI). For the isolation of an US PEDV strain, the day 4 intestinal contents from a pig inoculated with a stool sample from an Indiana farm with a PEDV outbreak in 2013 [33] were diluted in minimum essential medium (MEM) for 1:10 and filtrated using a 0.2 μm membrane filter. The prepared sample was inoculated to confluent Vero cells grown in the 6-well plates. The cells were then incubated at 37 °C in the presence of trypsin (1–2 μg/ml) in MEM. After 2 or 3 blind passages, apprarent cytopathic effects (CPEs) were observed in Vero cells. Viruses were further cloned with the limited dilution method and the cloned virus (P0) was confirmed as PEDV by IFA with an anti-PEDV antibody and the sequencing of spike (S) gene. The individual or pooled convalescent pig serum collected at 43 days after the virus inoculation from the previous pig challenge study [33] were used as the source of anti-PEDV antibody. The pooled sera collected prior to the virus inoculation were used as the negative control.

### Virus passages

The isolated PEDV (P0) was passaged in the presence of trypsin (1 μg/ml), elastase (1 μg/ml) or GCDCA (100 μM) in the media to generate PEDV KD, PEDV AA or PEDV 8aa, respectively. Viruses were further passaged in each condition for up to 120 passages. Every 3–5 passages, the titers of PEDV KD, PEDV AA or PEDV 8aa were determined by the TCID_50_ method (Reed-Muench method) in the presence of trypsin (1 μg/ml), elastase (1 μg/ml) or GCDCA (100 μM), respectively. These viruses were characterized using various experiments, including real time reverse transcription-quantitative polymerase chain reaction (RT-qPCR), immunofluorescent assay (IFA), Western blot analysis and inoculation to neonatal piglets. The PEDV N gene from PEDV P0 was amplified with primers of forward primer (aattCTCGAG atggcttctgtcagttttcagg) and reverse primer (aattGCGGCCGCttaatttcctgtgtcgaag) and amplied product was cloned in to the pSI vector (Promega, Madison, WI) using XhoI and NotI enzymes resulted in pSI-PEDV-N. The recombinant plasmid was transfected to Vero cells to express PEDV N to confirm the reactivity of antisera agaisnt the protein. Cell lysates were prepared at 24 h afte the tranfection for the Western blot analysis.

### Real-time RT-qPCR

PEDV RNA was extracted from the stool samples or cell culture following repeated freezing and thrawing using the RNeasy Kit (Qiagen, Valencia, CA) according to the manufacturer’s protocol. Real-time RT-qPCR was performed by using One-Step Platinum RT-qPCR kit (Invitrogen, Carlsbad, CA) using forward primer- 5′-GCTATGCTCAGATCGCCAGT-3′, reverse primer- 5′- TCTCGTAAGAGTCCGCTAGCTC-3′, and Probe- 5′−/56-FAM/TGCTCTTTG/ZEN/GTGGTAATGTGGC/3IABkFQ/−3′ targeting the N gene. The RT-qPCR amplification was performed in a Rotor-Gene Q (Qiagen) with the following conditions: 50 °C for 30 min and 95 °C for 5 min, then 40 cycles of denaturation at 95 °C for 15 s, annealing at 60 °C for 60 s and elongation at 72 °C for 30 s. The Ct values were converted to TCID_50_ equivalent/ml based on the equation derived from the standard curve generated with the serial dilution of cell culture-grown PEDV. The detection limit for this RT-qPCR was approximately 0.1 TCID_50_/ml.

### IFA

Confluent Vero cells grown in 96 well plates were infected with PEDV KD, PEDV AA or PEDV 8aa at a various MOI in the presence of trypsin, elastase or GCDCA, respectively. After 24 h of infection, cells were fixed with cold methanol. For IFA, the positive (against WT PEDV) or negative sera were added to each well, followed by the incubation with FITC-conjugated goat anti-swine IgG. The plates were then washed with PBS and visualized for fluorescence under a fluorescence microscope.

### Western blot analysis

Confluent Vero cells grown in 6 well plates were infected with PEDV KD, PEDV AA or PEDV 8aa in the presence of trypsin, elastase or GCDCA, respectively. Similarly, growth of PEDV 8aa with trypsin, elastase, GCDCA or without any addition were also monitored by Western blot analysis. Since PEDV 8aa grew to similar titers with or without FBS in the media, FBS was not added to the media. After 24 h post virus infection, cell lysates were prepared for SDS-PAGE (12% Tris-glycine gel), and proteins were transferred to nitrocellulose membranes. Membranes were probed with the positive (against WT PEDV) or negative pig sera followed by horseradish peroxidase-conjugated goat swine IgG. Proteins were visualized by chemiluminescence (Thermo Scientific). Concentrated (×100) PEDV KD, PEDV AA or PEDV 8aa were prepared by ultracentrifugation (100,000×g for 2 h in 30% sucrose cushion) and the concentrated viruses were serially diluted (1:5, 1:50 or 1:500) with PBS for the Western blot analysis.

### Determination of antibody titers by SN assay

The SN titers of the sera from pigs infected with WT PEDV [33] or of the serum samples from our pig studies were determined against PEDV KD, PEDV AA or PEDV 8aa P70. Each serum was serially diluted by 2-fold in MEM, and the diluted serum (50 μl each) was mixed with the same volume of media containing 200 TCID_50_ PEDV KD, PEDV AA or PEDV 8aa. The mixture was incubated in 37 °C for 30 min, then transferred to fresh Vero cells. The cells were further incubated in the presence of trypsin (1 μg/ml) for PEDV KD and PEDV AA or GCDCA (100 μM) for PEDV 8aa. Serum neutralization titers were determined by observing the appearance of CPE during the 3 days of incubation. Titers were determined as the reciprocal serum dilution of complete CPE inhibition.

### Immunization of inactivated PEDV 8aa in 4 weeks old pigs

To determine immunogenicity, BEI inactivated PEDV 8aa (P75) was inoculated to 4 week old pigs with an adjuvant. BEI was prepared by dissolving BEA in 0.175 M NaOH at 37 C for 1 h. The freshly prepared BEI was added to clarified virus culture (PEDV 8aa, P75, 7.5 log_10_ TCID50/ml) at final BEI concentrations of 1.0 mM. The mixture was incubated at 37 °C with continuous stirring for 24 h. The BEI was inactivated by adding 10% volume of 1 M sodium thio-sulphate in PBS. The inactivation of PEDV was confirmed by inoculating the samples to Vero cells. Approximately 4 week old pigs (*N* = 5) were immunized with intramuscularly (IM) in the neck with 2 ml of inactivated virus containing 20% adjuvant (Emulsigen®, MVP laboratories, Omaha, NE) with additional immunization at 3 weeks later. Final bleeding was done at 2 weeks after the last immunization. Control pigs (*N* = 3) were immunized with MEM and 20% adjuvant. Each serum sample was tested for SN titers against PEDV KD (P80), PEDV AA (P80) or PEDV 8aa (P75) as described above. The fluorescent foci neutralization assay (FFN) was also performed for each sample using a National Veterinary Services Laboratory (NVSL) reference isolate, USA/Colorado/2013 (CO/13) at the SDSU diagnostic laboratory to confirm the immunogenicity.

### Sequencing analysis of PEDV 8aa S gene

**Fig. 1:**
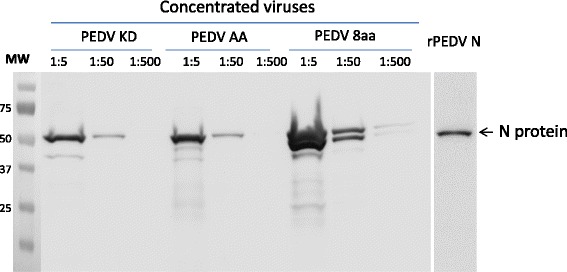
Western Blot analysis on the serial dilutions of the concentrated PEDV strains obtained in this study. PEDV KD (P120), PEDV AA (P103) or PEDV 8aa (P70) were concentrated by 100-fold with ultracentrifugation, and the concentrated viruses were diluted at 1:5, 1:50 or 1:500 for Western blot analysis. The pooled convalescent sera from US PEDV-infected pigs were used for the analysis. The PEDV N protein expressed in Vero cells (rPEDV N) by transfecting pCI-PEDV-N was included in the figure to confirm the reactivity to the protein by the pooled antisera

To investigate the potential underlying mechanism for enzyme-independent growth and/or reduced replication by trypsin of PEDV 8aa, the S gene of PEDV 8aa P0 (the early isolated US PEDV strain with trypsin in the media and P1 is the first virus passage number that was grown in GCDCA, Fig. 1), P70 and P105 were sequenced and compared to among them as well as those of the PEDV strains available in the GenBank (> 100 strains). The GenBank accession numbers for the S gene of PEDV 8aa P0 and P70 are KX834130 and KX834131, respectively. Because the S genes of PEDV P70 and P105 were identical, P70 S gene was deposited in GenBank.

### Trypsin pretreatment and time-of-addition assays

Trypsin pretreatment of viruses or cells and time-of-addition assays were performed to study the potential mechanism for the inhibition of PEDV 8aa (P70) replication by trypsin. PEDV KD (P70) was also included in this study as a control. For trypsin pretreatment of viruses, concentrated (10×) or un-concentrated PEDV 8aa or PEDV KD was incubated with mock-medium or trypsin (5 μg/ml) at 37 °C for 1 h. The mock- or trypsin-treated virus was then added to Vero cells in 6 well plates that were previously washed with cold PBS and the cells were then incubated at 4 °C for 1 h. After the incubation, cells were washed with cold PBS for 3 times, and cell lysates were prepared for real time RT-qPCR or Western blot analysis. The ct values from the real time RT-qPCR were converted to TCID_50_ values, as described below, and the TCID_50_ values were compared between the mock- or trypsin-treated PEDV 8aa or PEDV KD. In a separate experiment, PEDV 8aa pre-treated with mock-medium or trypsin was allowed to enter the Vero cells and replicate for 24 h. PEDV 8aa was incubated with trypsin (1 or 5 μg/ml) at 37 °C for 1 h and then fetal bovine serum (FBS) was added at 1% final concentrations to the mixture to neutralize trypsin. The virus was then inoculated to confluent Vero cells at an MOI of 10. The viral replication was monitored by virus titration or IFA staining at 24 h post inoculation. To determine if pre-treatment of cells by trypsin affects viral replication, confluent Vero cells were incubated with trypsin (5 μg/ml) at 37 °C for 1 h. After the incubation, the cells were washed 3 times with PBS, and PEDV 8aa or PEDV KD was added to the cell at an MOI of 10. The viral replication was determined by virus titration or IFA staining at 24 h post inoculation. The time-of-addition assay was also performed to determine which replication step of PEDV 8aa is inhibited by trypsin. Confluent Vero cells in 6 or 12-well plates were washed with PBS for 3 times and inoculated with PEDV 8aa (10 MOI). Mock-medium or trypsin (1 μg/ml) was then added at different time points: 0, 30 min, 1, 2, 4, 12 h after virus inoculation. Virus infected cells were further incubated for 24 h, and viral replication was assessed by viral titrations, IFA and Western blot analysis.

### Confocal microscopy

Using confocal studies for virus trafficking during entry, we demonstrated the endosomal escape of caliciviruses is critical for virus replication [34–36]. Because the addition of trypsin during up to 2 h post inoculation markedly inhibited virus replication, we examined the cellular trafficking of PEDV 8aa with or without trypsin using the same techniques. Vero cells were seeded onto Lab-Tek II CC2 chamber slide (Fisher Scientific, Pittsburgh, PA), treated with FBS and grown to 90% confluency. Confluent cells on the chamber slides were inoculated with mock-medium or PEDV 8aa (P70) at an MOI of 50 at 4 °C for 1 h. After mock-medium or trypsin (1 μg/ml) was added to the medium, the virus infected cells were further incubated in 37 °C for 30 min or 3 h before fixed for confocal microscopy. Cells were fixed in 4% paraformaldehyde (Sigma-Aldrich) in PBS (pH 7.4) at room temperature for 15 min, permeabilized with 0.1% Triton ×100 in PBS for 10 min at room temperature, washed three times with PBS, and incubated in blocking buffer (PBS containing 0.5% bovine serum albumin) for 15 min. Also fixed cells without permeabilization were prepared as a control. The cells were then incubated with the PEDV antibody (1:200) or Rab7 antibody (1:200) at 37 °C to probe PEDV or endosomes, respectively. After 2 h incubation at 37 °C, cells were washed three times with PBS and further incubated at 37 °C for 2 h with appropriate secondary antibodies. Cell nucleus was stained with sytox orange (0.5 μM in 0.9% NaCl). Coverslips were mounted in Prolong Gold antifade reagent (Molecular Probes), and the cells were scanned with a confocal microscope LSM 510 (Zeiss, Oberkochen, Germany) using a 100× oil-immersion objective. The images were analyzed by Image J software 1.47 (http://imagej.nih.gov/ij/) and merged images were prepared. The colocalization analysis of PEDV and Rab7 was performed using JACoP and colocalization-MBF plugins for ImageJ software. Single channel images were thresholded by Costes’ auto threshold method and the Manders split correlation coefficient for colocalization was then determined for each image.

### Determination of the level of attenuation of serially passaged PEDV in neonatal piglets

To determine the level of attenuation of serially passaged PEDV in neonatal piglets, we purchased PEDV-negative pregnant sows and their negative status with known enteric pathogens were confirmed with RT-qPCR. After farrowing of the sows, piglets were nursed by the sows in the farrowing crates for the duration of the virus attenuatioin study. The 1–4 day old piglets were orally inoculated with PEDV KD (P120), PEDV AA (P103), or PEDV 8aa (P40, P70 and P105). The virus inoculum was 1 × 10^6^ TCID_50_ per animal. After inoculation, clinical symptoms, survival and virus shedding (daily rectal swabs) were examined for up to 14 days. Stool samples were assessed by visual observation of rectal swabs and described as normal, loose stool or diarrhea (liquid with or without some solid content). Virus shedding was determined by real time RT-qPCR as described above. Serum samples were collected from each piglet weekly and tested for SN titers against PEDV 8aa.

## Results

### Cell culture adaptation of an US PEDV strain

Initial attempt to isolate the US PEDV strain required 2 or 3 blind passages with trypsin (1–2 μg/ml) until apparent CPE appeared in Vero cells. Virus growth in cell culture was confirmed by IFA using the PEDV antisera generated from the previous pig challenge study with a PEDV US strain [33]. The S gene of this PEDV isolate (designated as P0) showed a high sequence identity with those of other US PEDV strains (less than 10 amino acid residue differences from most of US PEDV strains detected or isolated during 2013–2014). To generate the PEDV KD, P0 was serially passaged in the presence of trypsin (1–2 μg/ml). After about 20 passages of PEDV, PEDV KD began to grow faster based on the apparent CPE appearance at 24 h post inoculation, leading to complete cell lysis by 48 h post inoculation. Syncytia formation was also observed in the cell monolayers. After passage number 20, PEDV KD titers consistently reached to 6.2–6.5 log_10_ TCID_50_/ml (Table 1).

**Table 1:** The adapted US PEDV strains passaged in three different culture conditions (trypsin, elastase and GCDCA) were designated as PEDV KD, AA or 8aa, respectively

PEDV KD		PEDV AA			PEDV 8aa		
Passage No	Titration with	Passage No	Titration with		Passage No	Titration with	
	Trypsin		Trypsin	Elastase		GCDCA	Trypsin
P4	5.1	P4	3.8	2.5	P4	2.5	3.1
P10	5.8	P10	5.2	4.1	P14	6.6	5.1
P20	6.3	P20	6.7	5.3	P17	7.5	2.2
P30	6.2	P30	6.5	5.1	P21	8.1	< 2
P40	6.5	P40	6.7	5.5	P40	8.1	< 2
P60	6.2	P60	6.8	5.3	P70	8.2	< 2
P120	6.3	P120	6.7	5.2	P120	8.1	< 2

The table shows PEDV titers (log_10_ TCID_50_/ml) which were determined in the presence of trypsin, elastase or GCDCA, as indicated

For PEDV AA, P0 was serially passaged in the presence of elastase (1–2 μg/ml). Apparent CPE began to appear after 2–3 passages of PEDV and PEDV AA titers gradually increased during the first 20 passages with syncytia formation. Interestingly, PEDV AA grew well with trypsin or elastase, which allowed virus titration to be performed using trypsin or elastase (Table 1). The titers of PEDV AA grown with elastase reached to 6.8 log_10_ TCID_50_/ml when titration was done with trypsin, which is slighly higher than those of PEDV KD (Table 1). However, when PEDV AA grown with elastase was titrated with elastase, the titers were approximately 10-fold lower than those determined with trypsin (Table 1). Nonetheless, antigenic masses of PEDV KD and PEDV AA strains were similar to each other when they were assessed with Western blot analysis after each virus was concentrated at the same ratio (Fig. 1).

**Fig. 2:**
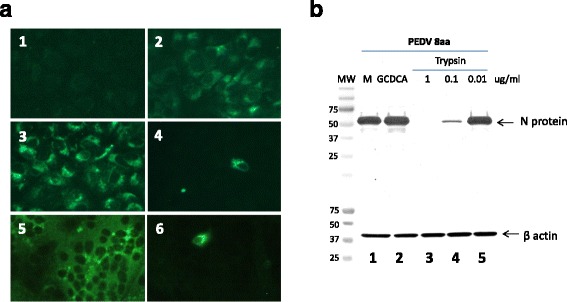
IFA and Western blot analysis of PEDV 8aa (P70) and PEDV KD grown in various culture conditions. **a** IFA: Vero cells were inoculated with mock-medium (Mock) (Panel 1), PEDV 8aa (P70) (Panels 2–4) or PEDV KD (P70) (Panels 5 and 6). PEDV 8aa-infected cells were further incubated in MEM containing mock (Panel 2), GCDCA (100 μM) (Panel 3) or trypsin (1 μg/ml) (Panel 4). Vero cells inoculated with PEDV KD (P70) (Panels 5 and 6) were further incubated in MEM containing trypsin (1 μg/ml) (Panel 5) or mock (Panel 6). At 24 h post virus infection, cells were fixed for IFA. **b** Western blot analysis: Vero cells were inoculated with PEDV 8aa (P70) at an MOI of 1, and virus infected cells were incubated with MEM only (M), GCDCA (100 uM) or various concentrations of trypsin (0.01 to 1 μg/ml) for 24 h. After 24 h of incubation, cell lysates were prepared for Western blot analysis for PEDV or β-actin

To generate PEDV 8aa, P0 was serially passaged with GCDCA (100 μM), but without adding any enzyme. PEDV 8aa began to efficiently grow after about 10 passages. Interestingly, syncytia formation was not observed in the cells infected with PEDV 8aa. Instead, extensive cell death (lysis) of cell monolayers occurred at approximately 40–48 h following virus infection. The virus titers of PEDV 8aa reached higher than 8.0 log_10_ TCID_50_/ml after passage number 20, which is substantially higher than those of PEDV KD or PEDV AA (Table 1). It is of note that, after 10 passages of initial adaptation to GCDCA, PEDV 8aa no longer required GCDCA for replication (Fig. 2a, Panels 2 and 3) and was able to grow in the presence or absence of FBS. The CPE progress or titers of PEDV 8aa grown in the presence with GCDCA were slightly faster or higher than those grown in mock (media only) or 2% FBS, respectively. It is noteworthy that the replication of PEDV 8aa became greatly inhibited by trypsin once viruses were completely adapted to grow with GCDCA after passage number 20 (Fig. 2a, Panel 4 and Table 1). The majority of the cells infected with PEDV 8aa with GCDCA or mock-medium were positively stained for PEDV (Fig. 2a, Panels 2 and 3). On the contrary, only few positive cells were observed by IFA staining when PEDV 8aa was grown with trypsin (1 μg /ml) (Fig. 2a, Panel 4). The inhibitory effects of trypsin on the replication of 8aa PEDV (passage number 21) was also confirmed by Western blot analysis in which the reduction of PEDV N protein levels was commensurate with trypsin concentrations in cell culture (Fig. 2b). On the contrary, PEDV KD (P70) efficiently replicated only in the presence of trypsin (Fig. 2a, Panels 5 and 6).

Replication of PEDV KD, PEDV AA or PEDV 8aa in cell culture was also determined by Western blot analysis, IFA and SN assay using the PEDV antisera. Figure 1 shows that the antisera from the pigs infected with a WT PEDV reacted to the tissue culture adapted viruses in Western blot analysis. The detection of N protein by the sera was confirmed with the recombinant PEDV N protein expressed from the plasmid (pCMV-PEDV N) carrying PEDV N gene under the CMV promoter. The N protein of PEDV 8aa appeared as two fragments in the Western blot analysis (Fig. 1) and this may be due to further modification (cleavage) of the N protein during replication as noted by Jaru-Ampornpan et al. [37]. When we determined the IFA and SN titers of the convalescent antisera against PEDV KD, PEDV AA or PEDV 8aa P70, the IFA titers against PEDV KD, PEDV AA or PEDV 8aa were 1255 ± 23, 1195 ± 41 or 1243 ± 31, respectively, and SN titers against PEDV KD, PEDV AA or PEDV 8aa were 48 ± 16, 43 ± 8.3, or 52 ± 4.7, respectively. The average SN titers of antisera immunized with the inactivated PEDV 8aa (with adjuvant) in 4 weeks old pigs against PEDV KD, PEDV AA or PEDV 8aa were 54.5 ± 8.0, 51.3 ± 6.9 or 57.6 ± 17, respectively. The average SN titers of the antisera (from the inactivated PEDV 8aa) against USA/Colorado/2013 (CO/13) was 52.8 ± 12.3 determined at the SDSU diagnostic laboratory. However, the sera from the control pigs immunized with MEM and 20% adjuvant did not show SN activity (<10) against these PEDV strains. These results indicate that PEDV AA, PEDV KD and PEDV 8aa retained comparable immunogenicity to wild type PEDV after cell-culture adaptation.

### Sequencing analysis of PEDV 8aa S gene

**Fig. 3:**
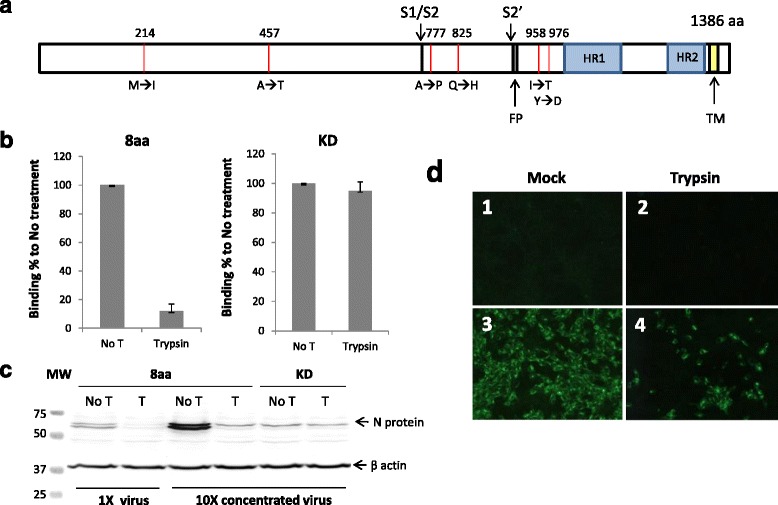
Mutations identified in the PEDV 8aa S gene and the effects of trypsin treatment on PEDV 8aa on virus binding or viral replication. **a** Amino acid changes in PEDV 8aa P70 or P105 from multiple sequence alignments with the parental PEDV 8aa P0 and >100 US PEDV strains. The S1/S2 junction (758/579), HR1 (aa 979–1088) and HR2 (aa 1239–1277) are indicated based on the literature [44, 45]. Fusion peptide (FP, aa 895–912) and transmembrane (TM) region are also shown in the schematic drawing. **b** and **c** Effects of trypsin treatment on PEDV 8aa or PEDV KD on virus binding. Unconcentrated or concentrated (10 ×) PEDV 8aa (P70) or PEDV KD (P70) was treated with trypsin (5 μg/ml) or mock-medium at 37 °C for 1 h and the treated virus was added to confluent Vero cells. The cells were then incubated in a cold room (4 °C) for 1 h and cell lysates were prepared for real time RT-qPCR (**b**) or Western blot analysis (**c**). The percentage of trypsin-treated viruses bound to the cells were compared to mock-medium treatment (No T, no treatment). **d** Effects of trypsin pre-treatment on PEDV 8aa on virus replication. Concentrated (10 ×) PEDV 8aa was pre-treated with trypsin (5 μg/ml) or mock-medium at 37 °C for 1 h, and added to confluent Vero cells. After incubation of the virus infected cells at 37 °C for 24 h, they were fixed for IFA staining for PEDV. Panels 1 and 2 show the cells incubated with trypsin or with mock-medium without virus infection, respectively. Panels 3 and 4 show PEDV 8aa grown in the cells with mock medium or trypsin respectively

The amino acid sequences of the S gene of PEDV 8aa P70 or P105 were identical. However, PEDV 8aa P70 (or P105) had 6 amino acid residue changes in the S gene when compared to P0 (Fig. 3a). Interestingly, the mutated 6 amino acids found in PEDV 8aa P70 or P105 are conserved in US PEDV strains (over 100 strains) whose sequences are available in the PubMed. Those conserved and mutated 6 amino acids, aa 214, 457, 777, 825, 958 and 976, are shown in Fig. 3a.

### Trypsin pretreatment and time-of-addition assays

Pretreament of cells with trypsin (5 μg/ml) did not affect the replication of PEDV 8aa or PEDV KD. However, pretreatment of PEDV 8aa with trypsin (5 μg/ml) led to a marked reduction (> 90%) in virus binding to the cells determined by real time RT-qPCR (Fig. 3b) or Western blot analysis (Fig. 3c) compared to the mock-medium treatment. In contrast, pretreatment of PEDV KD with trypsin (5 μg/ml) had little effect on the binding of PEDV KD to the cells (Fig. 3b and c). When PEDV 8aa pretreated with trypsin (1 or 5 μg/ml) for 30 min or 1 h was allowed to replicate in the cells for 24 h, viral replication was significantly reduced determined by viral titration (Table 2) or IFA staining compared to mock-medium treatment (Fig. 3d, Panels 3 and 4). Pretreatment of PEDV 8aa with trypsin (5 μg/ml) for 1 h resulted in over 10-fold reduction in viral titers compared to mock-medium treatment (Table 2).

**Table 2:** The effect of trypsin pretreatment of PEDV 8aa and time-of-addition assay with trypsin. Concentrated PEDV 8aa was used for this study

	Pre-treatment of virus with trypsin					Time-of-addition assay (post-inoculation)				
	Mock	1 μg/ml		5 μg/ml		Mock	1 μg/ml			
		30 min	1 h	30 min	1 h		0 h	2 h	4 h	12 h
Log_10_, TCID_50_/ml	7.23	6.59^*^	6.26^*^	6.34^*^	5.37^*^	7.10	1.60^*^	3.50^*^	6.00^*^	6.80

For trypsin pretreatment study, virus was incubated with trypsin at 1 or 5 μg/ml for 1 h at 37 °C and added to Vero cells. For time-of-addition assay, trypsin at 1 μg/ml was added to Vero cells infected with PEDV 8aa at the designated time points post infection. Viral titration was done at 24 h post infection for both pre-treatment and time-of-addition assay. Mock indicates treatment with mock-medium. * *P* < 0.05 compared to the control (Mock)

**Fig. 4:**
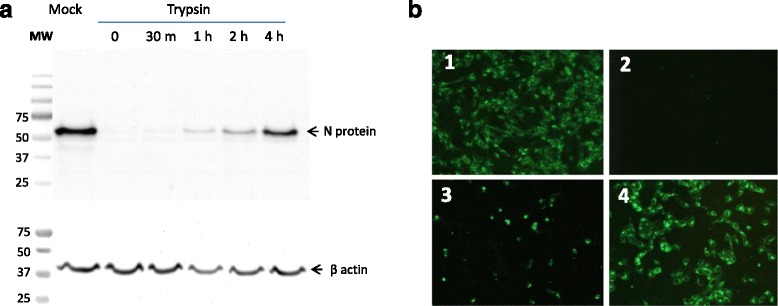
Replication of PEDV 8aa (P70) by the addition of trypsin at various time points determined by Western blot analysis or IFA. Confluent Vero cells were inoculated with PEDV 8aa (10 MOI) and mock-medium or trypsin (1 μg/ml) was added at different time points of 0, 30 min, 1, 2, or 4 h after virus inoculation. **a** Cell lysates were prepared at 24 h post inoculation for Western blot analysis with antibody against PEDV or β-actin. **b** Cells were fixed at 24 h post inoculation for IFA staining. Panel 1 shows the cells with mock-medium treatment. Panels 2–4 show the virus-infected cells where trypsin was added at 0, 1 or 4 h post virus inoculation, respectively

The time-of-addition assay showed that the addition of trypsin during 0 to 2 h post inoculation markedly inhibited PEDV 8aa replication determined at 24 h (Fig. 4a and b and Table 2). When trypsin was added at the same time of PEDV 8aa inoculation (0 h) or 1 h post inoculation, greater than 10^5^- or 10^3^-fold reduction in viral titers was observed compared to mock-medium treatment, respectively (Table 2). Both Western blot analysis and IFA results correlated with the reduced viral titration (Fig. 4a and b). However, there was no significant difference in viral replication when trypsin was added at 4 h post inoculation (Fig. 4a and Fig. 4b, Panel 4).

### Confocal studies

**Fig. 5:**
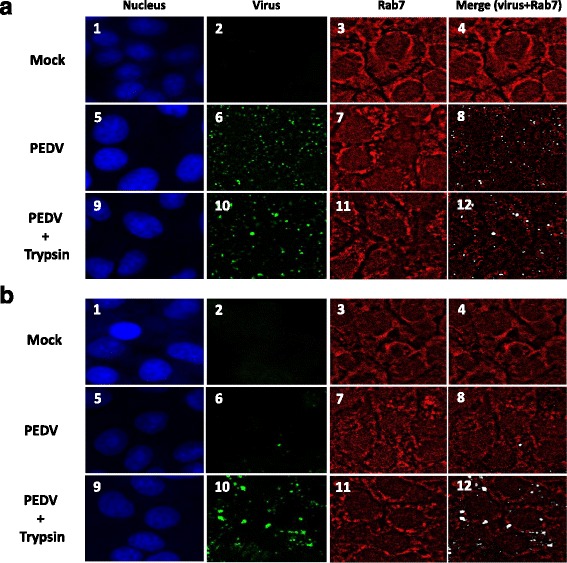
Confocal microscopic study on the effects of trypsin on the entry of PEDV 8aa (P70) into the cells. (a and b) Vero cells were inoculated with mock-medium (Panel 1 to 4) or PEDV 8aa (50 MOI) and incubated at 4 °C for 1 h, then mock-medium (Panels 5 to 8) or trypsin (Panels 9 to 12) were added to the cells. The cells were further incubated at 37 °C for 30 min (**a**) or 3 h (**b**) and then fixed for confocal microscopy. Fixed cells were probed with sytox orange (blue) for nucleus, anti-Rab7 antibody (red) for the endosomes or anti-PEDV antibody (green). Merged images of viruses and the endosomes (white) were prepared (Panel 4, 8, 12 in **a** and **b**)

**Fig. 6:**
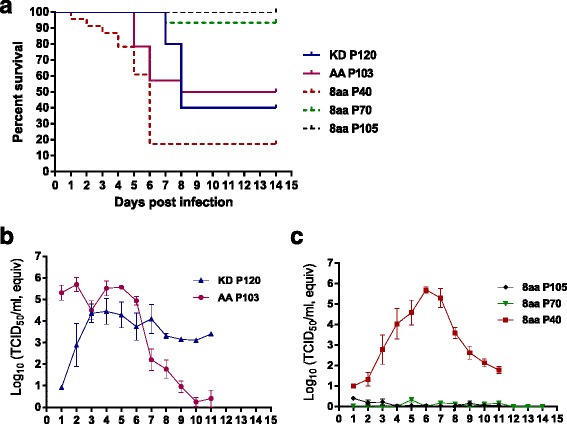
Survival curves and virus shedding of nursing piglets inoculated with PEDV KD, AA, or 8aa at various passage numbers. **a** Survival curves of 1–4 day old piglets inoculated with PEDV KD P120 (KD P120), PEDV AA P103 (AA P103), PEDV 8aa P40 (8aa P40), PEDV 8aa P70 (8aa P70) or PEDV 8aa P105 (8aa P105). **b** and **c** Virus shedding of piglets inoculated with each strain. Daily rectal swabs were collected and virus shedding was determined by real-time RT-qPCR. The Ct values were converted to TCID_50_/ml equivalent values (log_10_). The mean and the standard errors are shown

Confocal studies for virus trafficking during the virus entry demonstrated that PEDV 8aa was not able to escape from endosomes in the presence of trypsin. At 30 min post inoculation of PEDV 8aa, there was no noticeable difference in the fluorescence signals for virus (green) in the cells with or without trypsin (Fig. 5a, panels 6 and 8). At 3 h post inoculation, fluorescence signals for virus disappeared in the cells without trypsin, (Fig. 5b, Panel 6), suggesting successful PEDV release to the cytoplasm at the time point. However, in the presence of trypsin, fluorescence signals still remained (and increased) in the cells (Fig. 5b, Panel 10). The increased signals in Fig. 6b, Panel 10 suggest accumulation of PEDV in the compartments without releasing to cytoplasm at that time point. Fluorescence signals remained in the cells even at 24 h post inoculation in the presence of trypsin (data not shown). Fluorescence signals for virus (green) co-localized (white) with the endosomal marker Rab7 (red) with Manders split colocalization coefficient of >0.90 (Fig. 5a, Panels 8 and 12 and Fig. 5b, Panels 8 and 12). These results suggest that PEDV 8aa failed to escape from the endosomal compartments in the presence of trypsin, while viruses in the trypsin-free media were able to exit the endosomal compartments to the cytoplasm and to initiate virus replication.

### Pathogenicity of serially passaged PEDVs in neonatal piglets

The results of the animal studies to determine the level of attenuation of PEDV KD, PEDV AA, and PEDV 8aa (P40, P70 and P105) in neonatal piglets are summarized in Table 3. Fatality varied among groups from 0 to 100% (Table 3, Fig. 6a). The onset and duration of symptom (diarrhea) and virus shedding also differed among groups (Table 3, Fig. 6b and c).

**Table 3:** Summary of pathogenicity (attenuation) studies of PEDV KD (P120), PEDV AA (P103) or PEDV 8aa (P40, P70 or P105) in 1–4 day old piglets

Group	Inoculum	Age at inoculation (numbers)	No. of death (fatality %)	Clinical symptoms	Virus shedding
1	PEDV KD P120	2-day old (N = 5)	3/5 (60%)	Diarrhea: 3–9 DPI	Started at 2 DPI
2	PEDV AA P103	2-day-old (*N* = 14)	7/14 (50%)	Diarrhea: 2–9 DPI	Started at 1 DPI
3	PEDV 8aa P40	2 or 4-day old (*N* = 24)	20/24 (83.3%)	Diarrhea: 3–8 DPI	Started at 2 DPI
4	PEDV 8aa P70	1-day-old (*N* = 15)	1/15 (6.7%) 1 died (non-PEDV related)	Loose stool at 1–2 DPI	Limited virus shedding
5	PEDV 8aa P105	1- or 2-day old (*N* = 16)	0/16 (0%)	No diarrhea	Limited virus shedding

After virus inoculation, piglets were observed daily for clinical signs and rectal swabs were collected daily for the determination of virus shedding for up to 14 days post inoculation. DPI, days post infection

In PEDV KD P120 group, 2-day old piglets were inoculated with the strain, and three out of five piglets died during 6 to 8 days post-infection (DPI) (60% fatality) (Table 3, Fig. 6a). Virus shedding and diarrhea started at 2 and 3 DPI, respectively. Fecal viral titers reached to 4.5 log_10_ TCID_50_ equivalent/ml at 4 DPI and remained above 3–4 log_10_ TCID_50_ equivalent/ml until 11 DPI (end of observation)(Fig. 6b). In PEDV AA P103 group, 2-day old piglets were inoculated with PEDV AA P103. During 5 to 8 DPI, 7 out of 14 piglets died (50% fatality) (Table 3 and Fig. 6a). Virus shedding and diarrhea started at 1 and 2 DPI, respectively. Fecal viral titers reached to 5.2 and 5.5 log_10_ TCID_50_ equivalent/ml at 1 and 2 DPI, respectively, and remained higher than 4–5 log_10_ TCID_50_ equivalent/ml until 6 DPI. Fecal virus titers gradually reduced to lower than 1 log_10_ TCID_50_ equivalent/ml after 9 DPI (Fig. 6b).

Three groups were inoculated with three different passage numbers (P40, P70 and P105) of PEDV 8aa. They were comprised of piglets of 1 day-old (P70), 1–2 day old (P105) or 2 or 4 day old (P40) at the time of virus inoculation (Table 3). The piglets infected with PEDV 8aa P40 sustained high fatality during 1–6 DPI (83.3% fatality) with high viral shedding and diarrhea starting at 2 and 3 DPI, respectively (Fig. 6a and c). In this group, the average fecal viral titers reached to 5.7 log_10_ TCID_50_ equivalent/ml at 6 DPI (Fig. 6c). However, inoculation of piglets with PEDV 8aa P70 or P105 led to only one (6.7% fatality) or no death, respectively (Table 3, Fig. 6a). One piglet died after inoculation of PEDV 8aa P70 does not seem to be related to PEDV as there was no clinical symptom or virus shedding. In some animals infected with PEDV 8aa P70, loose stool was observed at 1 and 2 DPI, which resolved after 2 DPI, and limited virus shedding (< 1 log_10_ TCID_50_ equivalent/ml) was observed (Fig. 6c). In the PEDV 8aa P105 group, there was no loose stool or diarrhea with limited virus shedding with <1 log_10_ TCID_50_ equivalent/ml (Fig. 6c). The survival rates of the piglets and viral shedding/diarrhea indicate that PEDV 8aa became greatly attenuated after passage number 70.

## Discussion

Until recently, most PEDV strains circulating in the Asia belonged to the genogroup 1 and, in those countries, genogroup 1 PEDV MLVs have been used for the control of PEDV [26–29]. The MLVs (including 83P-5 and DR13 strain) were generated by continuous passages of wild-type viruses in Vero cells (> 100 times) and their attenuation in piglets and sows and induction of a robust immune response in sows were previously demonstrated [38]. The US PEDV strains have approximately 10% genetic diversity in the S1 gene compared to that of the genogroup 1 and the US PEDV strains are phylogenetically grouped in subgroup 2a [4, 11, 30]. Although there is a varying degree of cross-reactivity and cross-neutralization among some US PEDV strains and the genogroup 1 PEDV prototype strain CV777 in in vitro assays [39], the protective efficacy of the genogroup 1 PEDV MLV against the genogroup 2 PEDV strains has not been reported. Considering the genetic and antigenic differences in S1 between the genogroups, effective control of US PEDV outbreaks is expected to require both genogroups 1 and 2 PEDV vaccines. For both killed vaccines or MLV production, generation of high-titered PEDV in cell culture is important for vaccine production [40]. However, propagation of PEDV to high titers (>8 log_10_ TCID_50_/ml) is not easily achieved. In this study, to generate US PEDV strains with properties suitable for vaccine production, we investigated the effects of various selective pressures on viral growth characteristics by serially-passaging an US PEDV isolate in cell culture. Since trypsin is typically required for isolation and propagation of PEDV in cell culture, we first passaged PEDV in the presence of trypsin to generate PEDV KD. Although PEDV can be ultimately adapted to grow in cell culture without trypsin, exemplified by DR13 or PEDV 8aa, PEDV is the only known coronavirus which requires trypsin in the medium for isolation and replication in cell culture. Using trypsin during viral passages, we generated PEDV KD that induces typical, extensive cell fusion with syncytium formation and its titers are similar to those reported by other groups [11, 12, 40]. In addition to PEDV KD adapted to grow in the presence of trypsin, we generated PEDV AA and 8aa that do not require trypsin for viral propagation. PEDV AA was obtained by passaging the WT US strain with elastase in cell culture. Elastase is a proteolytic enzyme produced by pancreas or neutrophils and involved in nutrient digestion in the intestines or catalyzing the breakdown of elastin or bacteria during inflammation in the lung. Elastase recognizes the carboxyl groups of small hydrophobic amino acid such as glycine, alanine and valine for proteolytic activity, while trypsin recognizes basic amino acids such as arginine or lysine. Interestingly, the resulting PEDV AA retains the ability to grow either in the presence of elastase or trypsin and induces extensive cell fusion similar to that by PEDV KD. PEDV 8aa was generated by serially passageing the WT PEDV in the presence of bile acid. We and other groups have previously shown that bile acids influence the replication of various viruses that propagate in the bile-rich organs such as liver and intestines, which include porcine enteric calicivirus, hepatitis B and C viruses and rotaviruses [41–43]. Bile acids are synthesized in the liver, stored in the gallbladder and released into the duodenum, where they play important roles in absorption of nutrients, lipid and cholesterol metabolism and also imunomodulation [44]. Interestingly, PEDV 8aa gradually lost its ability to grow in the presence of trypsin during adaptation to bile acid (Table 1 and Fig. 2). The other change observed concurrently is that PEDV 8aa induced extensive cell death (lysis) without cell fusion. The role of GCDCA in the replication of PEDV is unknown, however, it was previously reported that serial passages of PEDV in cell culture (> 100 passages) led to generation of viruses that grow in trypsin-free media (DR13 vaccine strain). Therefore, it can be speculated that GCDCA may facilitate the adaptation of the virus to trypsin-free growth during the early phases of adaptation. However, once PEDV are fully adapted to the absence of these extraneous supplement in media, GCDCA does not substantially promote or reduce viral replication in cell culture. Importantly, PEDV KD, AA and 8aa retained immonogenicity (IFA and SN) to the pooled sera generated against the WT PEDV. Since PEDV 8aa consistently grows to high titers in FBS or enzyme-free media, this adapted virus would offer advantages in cell-culture based production of viruses for vaccines.

The sequence analysis of the S gene of PEDV 8aa P70 and P105 revealed that they have six unique mutations compared to the parental P0 virus and other reported US PEDV strains. Four out of six mutations are located near the S1/S2 junction and the fusion peptide (Fig. 3a). Recently, Wicht et al. [45] demonstrated that the S protein determines trypsin-dependency of PEDV replication and that trypsin-enhanced PEDV entry was mapped to the region from S1/S2 junction to HR1 by using a recombinant PEDV carrying the S protein of CV777 (trypsin-dependent strain) or DR13 (trypsin-independent strain). In this study, trypsin pretreatment and the time-of-addition assays using PEDV 8aa showed that trypsin prevents viral attachment to the host cells and inhibits the early phase of virus replication, including virus entry (Figs. 3 and 4, Table 2). The confocal microscopy study demonstrated that PEDV 8aa failed to exit the endosomal compartments for viral replication in the presence of trypsin (Fig. 5). The PEDV 8aa was detected in the endosomal compartments even after 24 h post inoculation in the presence of trypsin (data not shown). We have previously reported that endosomal uptake and subsequent endosomal escape of caliciviruses (porcine enteric calicivirus and murine norovirus) is critical for virus replication by showing that virus replication was completely or significantly reduced when the endosomal escape event was blocked [34–36]. Based on our results, it can be speculated that those mutations found in the S protein of PEDV 8aa may be associated with trypsin-independent viral growth as well as trypsin-mediated inhibition of virus entry to the host cells. Currently, we are investigating the roles of the mutations in both trypsin independent growth and viral entry inhibition.

In the neonatal piglets, PEDV KD and PEDV AA at the passage number over 100 still retained virulence (Fig. 6), and further passages may be required for attenuation of these strains. It was previously shown that about 100 passages of genogroup 1 PEDV in Vero cells resulted in attenuation in piglets, which are 83P-5 and DR13 MLVs [26, 29]. It is possible that genogroup 2 PEDV may require more serial passages in cell culture to become attenuated in piglets, as shown in a recent report where attenuation of a US PEDV was achieved with at least 160 passages in cell culture [46]. In this study, PEDV 8aa P70 and P105 were found to be fully attenuated in the neonatal piglets (Fig. 6) and substantial attenuation seems to have occurred between P40 and P70 (Fig. 6a and c). These results suggest that growth inhibition of PEDV 8aa by trypsin or elastase may be the key for the fully attenuated phenomenon of PEDV 8aa in piglets. Of note, it was previously suggested that ORF3 protein is also involved in the PEDV attenuation [47] but the sequencing results of ORF3 gene from PEDV 8aa P70 or P105 showed no mutation in the ORF3 in these strains compared to the earlier passage numbers. We plan to investigate the mechanism of attenuation of PEDV 8aa P70 and P105 by conducting various experiments.

## Conclusions

We showed that US PEDV strains (genogroup 2) adapted to various culture conditions have different growth characteristics. One (PEDV 8aa) of the generated strains is able to replicate in cells without any protease. Interestingly, replication of PEDV 8aa is severely reduced by the presence of trypsin and this correlates with impaired virus entry into the cells. Because PEDV 8aa P70 (and P105) was significantly attenuated in neonatal piglets, this strain may warrant further investigation as a candidate for MLV for the control of emerging US PEDV. Further investigation would include vaccination and passive protection study using PEDV 8aa in pregnant sows and neonatal piglets.

## Abbreviations

BEA: Bromoethylamine hydrobromide
BEI: Binary ethyleneimine
CPE: Cytopathic effect
DMEN: Dulbecco’s minimal essential medium
FBS: Fetal bovine serum
GCDCA: Glychenodeoxycholic acid
IFA: Immunofluorescent assay
MEM: Minimum essential medium
MLV: Modified live attenuated vaccine
PEDV: Porcine epidemic diarrhea virus
RT-qPCR: Real time-quantitative polymerase chain reaction
S: Spike
SN: Serum neutralization
TCID50: Tissue culture infectious dose 50
TPCK: L-1-tosylamide-2-phenylethyl chloromethyl ketone
